# Supplementary material for: Sleep Characteristics in Dogs; Effect on Caregiver-Reported Problem Behaviours
Source: Animals (Basel). 2022 Jul 8;12(14):1753. doi: 10.3390/ani12141753 (PMC9312228; doi:10.3390/ani12141753)
Supplement: Supplementary file 1 [file animals-12-01753-s001.zip › animals-1759246-supplementary.pdf]

Behavioural Referrals Ltd veterinary practice
a referral service in behavioural medicine

Do our dogs sleep enough?

I am Carrie Tooley, a veterinary surgeon working in referral Behavioural Medicine. I am researching sleep in pet dogs in the UK and I invite anyone living with a companion dog to complete this questionnaire. You could win a Ginger and Brown's Tail Mail prize box.

I intend to establish how much sleep the average pet dog gets in our UK households, and whether the quality and quantity of sleep our dogs achieve influences their behaviours.

I have designed a short questionnaire, which should take less than 10 minutes to complete. Your input will be very much appreciated.

The questionnaire will be completed anonymously, and your completion cannot lead to your identification. However, if you are willing to answer the questions about yourself and the area you live in, this will help me to identify any associated trends. This information will not be used to link back to you.

You may stop the questionnaire at any point, without clicking "submit" and any data entered to this point will not be retained. At the point of clicking "submit" your data will be entered and anonymised, and I will be unable to remove this data from the study.

By submitting answers to this questionnaire, you consent to your responses being used in this study and future studies (anonymously).

If you have any questions about this questionnaire, or experience any difficulties in completing it, please do get in touch with me, Carrie Tooley (carrie@brvp.co.uk).

I would like to take this opportunity to thank you very much for taking the time to be involved in this important research, and help us understand this significant topic.

Best Wishes,

Dr Carrie Tooley BSc BVetMed MSc PgCertVE FHEA MRCVS
RCVS Advanced Practitioner in Companion Animal Behaviour
Resident of the European College of Animal Welfare and Behavioural Medicine (BM)

Supervisor: Dr Sarah Heath BVSc PgCertVE DipECAWBM(BM) CCAB FHEA FRCVS
RCVS Veterinary Specialist in Behavioural Medicine
EBVS® European Veterinary Specialist in Behavioural Medicine

If you own **more than one dog,** please choose **the dog whose name comes first alphabetically** and respond regarding that individual throughout the questionnaire. You may answer the whole questionnaire again for another dog, but each questionnaire answer sheet should represent answers from one individual dog throughout, and each dog should only be the subject of one questionnaire answer sheet.

About this dog and your household:

Please complete the following information about your dog:

1. Age:
2. Sex: M/F
3. Neuter status: neutered/not neutered
4. Breed:
5. How long your dog has lived with you:
6. How many human family members normally live with this dog?
7. How many other canine family members normally live with this dog?
8. Does this dog live with any other (non-human, non-canine) animals? (If yes, please specify):

Questions about this dog's sleep:

If this dog's sleep patterns have changed significantly due to Covid-19 associated changes in routine, please answer this section in relation to this dog's previous long term sleep patterns.

Q9
To the best of your knowledge, how many hours sleep does your dog normally achieve whilst you are in bed?

- Up to 2 hours
- 2 – 3hr 59min
- 4 – 5hr 59min
- 6 – 7hr 59min
- 8 – 9hr 59min
- 10 – 11hr 59min
- 12 hours or more

Q10
To the best of your knowledge, how many hours sleep does your dog normally achieve whilst you are out of bed?

- Up to 2 hours
- 2 – 3hr 59min
- 4 – 5hr 59min
- 6 – 7hr 59min
- 8 – 9hr 59min
- 10 – 11hr 59min
- 12 hours or more

Q11
What signs indicate to you that your dog is asleep (tick all which apply)?

- They breath more slowly
- They snore
- Their eyes are shut
- They are staying still
- They twitch or vocalise as though dreaming
- They go floppy/have relaxed muscle tone
- They are unresponsive to stimuli that would normally cause excitement
- Other (please give details):

If this dog ever sleeps in a room *without another pet present*, please answer questions 12, 13 and 14 with reference to the times they sleep without your other pet(s).

If this dog *always* sleeps in a room *with* another pet, please move straight to question 15.

Q12
Please tick the box correlating with your opinion on each of the following statements:

	Always	Usually	Sometimes	Rarely	Never	Unsure/ Not applicable
This dog chooses to sleep in a room with a human family member present.	○	○	○	○	○	○
This dog chooses to sleep without a human present.	○	○	○	○	○	○
This dog has free choice on whether to sleep with or without human company.	○	○	○	○	○	○
Other: this dog has varying preferences regarding sleeping with human company (if so, please give details).	○	○	○	○	○	○

Details (FREE TEXT ANSWER):

Q13
Please tick the box correlating with your opinion on each of the following statements:

**If you were to enter or leave, the room that this dog is asleep in, would they:**	Always	Usually	Sometimes	Rarely	Never	Unsure/ Not applicable
Stay asleep.	○	○	○	○	○	○
Wake and follow you.	○	○	○	○	○	○
Wake and assess what you were doing before deciding whether to react.	○	○	○	○	○	○

Their response varies (please give details). (FREE TEXT ANSWER):

Q14
Please tick the box correlating with your opinion on each of the following statements:

**If this dog is asleep and a noise occurs outside your home which you are easily able to hear, this dog would:**	Always	Usually	Sometimes	Rarely	Never	Unsure/ Not applicable
Stay asleep.	○	○	○	○	○	○
Wake and find/follow you.	○	○	○	○	○	○
Wake and assess what is happening before deciding whether to react.	○	○	○	○	○	○
Wake and react to the noise regardless of your location or reaction.	○	○	○	○	○	○

Their response varies (please give details). (FREE TEXT ANSWER):

Q15
If you would like to add any additional information about this dog’s sleep habits, please use this space: FREE TEXT ANSWER

A question about this dog's behaviour:

Q16
On a scale of 0 (this dog is perfect and shows no problem behaviours) to 10 (this dog behaves in a way I cannot tolerate), please give your opinion on the severity of the behaviour this dog shows:

Q17
Please briefly list this dog's unwanted behaviours:

A question about the impact of Covid-19:

Q18
Did Covid-19 "lockdown" restrictions significantly alter the routine of the humans OR this dog in your household?

○ No (please move straight to question 21)

○ Yes – please tick the box correlating with your opinion on each of the following statements:

Q19

	Greatly increased	Increased	No change noted during "lockdown"	Decreased	Greatly decreased	Unsure/ Not applicable
During "lockdown"…						
…the amount of human activity in my house…	○	○	○	○	○	○
… the time this dog was alone in the house…	○	○	○	○	○	○
…the number of human family members living at home …	○	○	○	○	○	○
… the stress level amongst the human family members …	○	○	○	○	○	○
…the time that family members were working from home, so they were present, but not actively giving this dog attention…	○	○	○	○	○	○
…the amount of child-related activity in this household …	○	○	○	○	○	○
…the amount this dog was walked…	○	○	○	○	○	○
…the amount of human attention this dog had…	○	○	○	○	○	○
…the amount this dog slept ….	○	○	○	○	○	○
…this dog's quality of life…	○	○	○	○	○	○
…the problem behaviours this dog displayed…	○	○	○	○	○	○

Q20
If you would like to add any additional information about the impact of "lockdown" on this dog and its behaviour, please use this space: FREE TEXT ANSWER

Thank you for sharing your experiences with me. The survey is almost complete. Please could you now answer these final demographic questions.

Q21

Please select your age:

○ < 19 ○ 20-29 ○ 30-39 ○ 40-49 ○ 50-59 ○ 60-69

○ 70-79 ○ 80+ ○ Prefer not to say

Q22
Please select your gender

○ Female ○ Male ○ Other ○ Prefer not to say

Q23
Which UK county do you live in?

Q24
Would you class the level of ambient noise around your house as:

- Very quiet
- Quiet
- Neither quiet nor noisy
- Noisy
- Very noisy

Q25 Thank you for reaching the end of the survey. If you would like to be entered into the prize draw to win a Ginger and Brown's Tail Mail prize box, please leave your name and email address below.

Please note, these details will only be used in relation to the prize draw.

FREE TEXT ANSWER

If you feel you need help with a behavioural problem or behavioural change that your dog is showing, you should contact your vet for a health check. More information is available on the Fellowship of Animal Behaviour Clinicians website or on the Behavioural Referrals Veterinary Practice website.

www.fabclinicians.org/find-a-behaviourist

www.behaviouralreferrals.co.uk

Q26 If you wish to receive a summary of the results of this questionnaire, please leave your name and email address:

- ○ Please use the details provided for the draw.
- ○ Please use a different name and address: FREE TEXT ANSWER
